# The porcine islet-derived organoid showed the characteristics as pancreatic duct

**DOI:** 10.1038/s41598-024-57059-1

**Published:** 2024-03-16

**Authors:** Naoaki Sakata, Gumpei Yoshimatsu, Ryo Kawakami, Kazuaki Nakano, Teppei Yamada, Akihiro Yamamura, Hiroshi Nagashima, Shohta Kodama

**Affiliations:** 1 Department of Regenerative Medicine and Transplantation, Faculty of Medicine, Fukuoka University, 7-45-1 Nanakuma, Jonan, Fukuoka, Fukuoka 814-0180 Japan; 2 Center for Regenerative Medicine, Fukuoka University Hospital, 7-45-1 Nanakuma, Jonan, Fukuoka, Fukuoka 814-0180 Japan; 3 Meiji University International Institute for Bio-Resource Research, 1-1-1 Higashimita, Tama, Kawasaki, Kanagawa 214-8571 Japan; 4 Department of Surgery, Tohoku University Graduate School of Medicine, 1-1 Seiryomachi, Aoba, Sendai, Miyagi 980-0872 Japan

**Keywords:** Organoid, Pancreas, Pig, Islet, Pancreatic duct, Transplantation, Translational research, Islets of Langerhans, Pancreas, Cell transplantation

## Abstract

Organoid is a tissue-engineered organ-like structure that resemble as an organ. Porcine islet-derived organoid might be used as an alternative donor of porcine islet xenotransplantation, a promising therapy for severe diabetes. In this study, we elucidated the characteristics of porcine islet organoids derived from porcine islets as a cell source for transplantation. Isolated porcine islets were 3D-cultured using growth factor-reduced matrigel in organoid culture medium consist of advanced DMEM/F12 with Wnt-3A, R-spondin, EGF, Noggin, IGF-1, bFGF, nicotinamide, B27, and some small molecules. Morphological and functional characteristics of islet organoids were evaluated in comparison with 2D-cultured islets in advanced DMEM/F12 medium. Relatively short-term (approximately 14 days)—cultured porcine islet organoids were enlarged and proliferated, but had an attenuated insulin-releasing function. Long-term (over a month)—cultured islet organoids could be passaged and cryopreserved. However, they showed pancreatic duct characteristics, including cystic induction, strong expression of *Sox9*, loss of PDX1 expression, and no insulin-releasing function. These findings were seen in long-term-cultured porcine islets. In conclusion, our porcine islet organoids showed the characteristics of pancreatic ducts. Further study is necessary for producing porcine islet-derived organoids having characteristics as islets.

## Introduction

Organoid is defined as tissue-engineered organ-like structures which recapitulate many characteristics of in vivo organ^1,2^. It is an in vitro a three-dimensional (3D) cellular cluster derived from primary tissue or pluripotent stem cells with the capabilities of self-renewal, self-organization, and similar organ functionality^3^. Since today, Various organoids which harbor the characteristics of each organ including brain^4^, lung^5^, liver^6^, thyroid^7^ have been widely developed. Regarding pancreatic islet, some groups succeeded to develop islet organoid which ameliorated diabetic animals. For example, Wang and colleagues developed pancreatic islet organoids by 3D co-culture of islet progenitors and endothelial cells^8^. Yoshihira and colleagues also developed islet organoids using human iPS cell-derived insulin-producing cells, human adipose-derived stem cells, and human umbilical vein endothelial cells^9^. Therefore, islet organoids might be used as alternative donors for islet transplantation, a promising therapy for patients with severe diabetes mellitus (DM).

Recently, we try to promote porcine islet xenotransplantation, because islet transplantation has major hurdles because of limited donor supplies^10^. Adult pigs are a representative alternative donor to humans. Recent progress in gene-editing technology has permitted the production of porcine-specific antigen and porcine-derived pathogen-free pigs^11,12^ that will support the feasibility of xenotransplantation without rejection and donor-induced adverse events. For the success of this therapy, it requires large numbers of porcine islets with high insulin secretion. However, porcine islet isolation is technically difficult because of the vulnerability of porcine islets^13,14^. Furthermore, long-term cultured porcine islets are hardly to preserve in insulin secretion^15^. Fresh islets are recommended for transplantation. That means, it might be difficult to prepare sufficient porcine islet yield for normoglycemia by one time islet isolation. For overcoming this limitation, we have tried to produce islet organoids derived from porcine islets. Some studies have revealed that organoids can be cultured for a long term, proliferated and cryopreserved^16–18^. If the islet organoids harbor similar characteristics to porcine islets, they can be used as considerable donor of transplant therapy for DM. However, detailed characteristics of the porcine islet-derived organoids have not been fully discussed.

In this study, we tried to elucidate the characteristics of islet organoids derived from porcine islets.

## Results

### Porcine islet characteristics change under relatively short-term organoid culture

**Figure 1:**
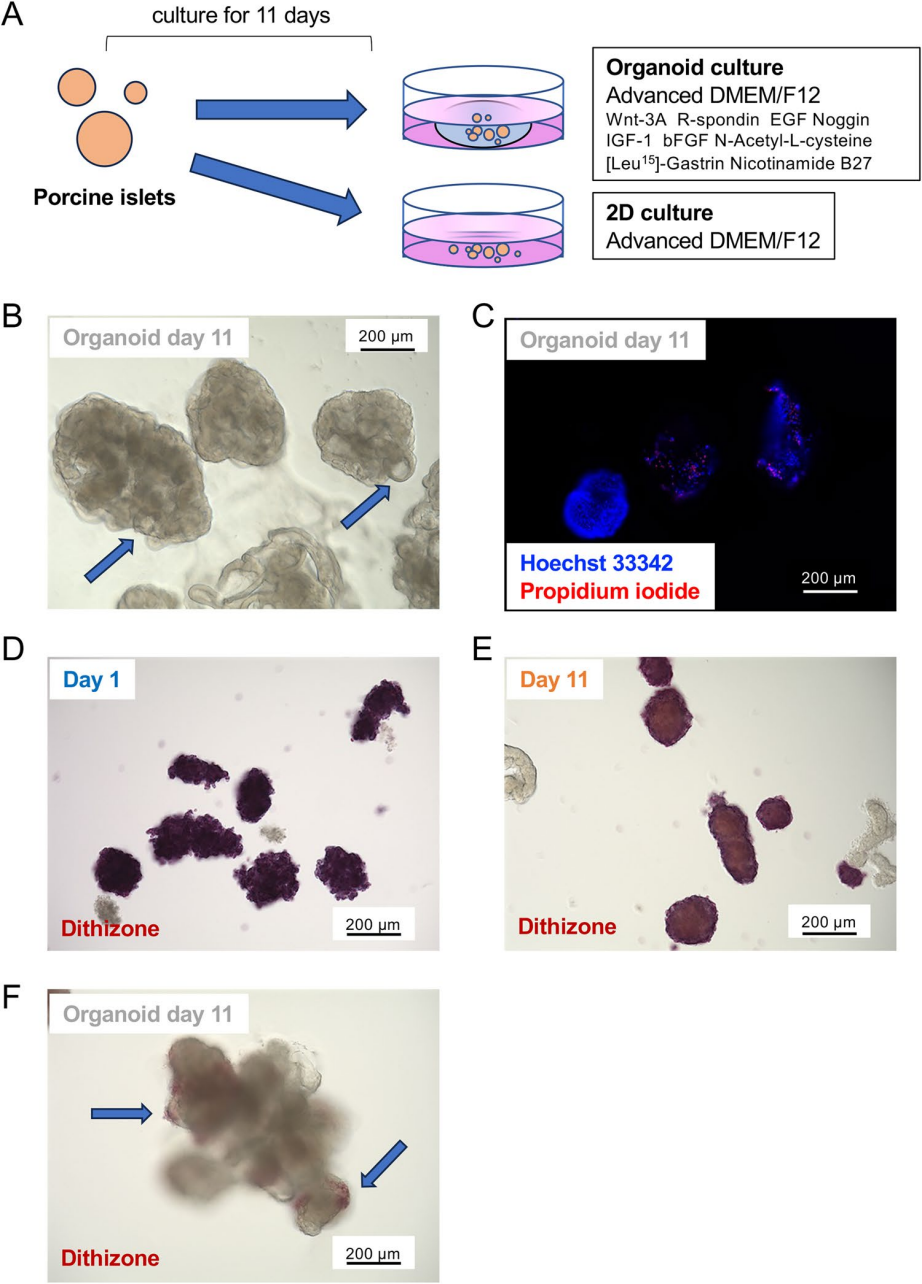
Characteristics of porcine islet organoids on day 11. (**A**) Scheme of islet organoid formation using porcine islets. The porcine islets were cultured in advanced DMEM/F12 with Wnt-3A, R-spondin, EGF, Noggin, IGF-1, bFGF, N-acetyl-l-cysteine, [Leu^15^]-gastrin, and nicotinamide (organoid culture) or advanced DMEM/F12 without supplements (2D culture). (**B**) Islet organoids on day 11. Blue arrows indicate cystic components in the organoids. (**C**) Islet organoids stained with Hoechst 33342 (blue) and propidium iodide (red) to assess viability. (**D**) Overnight-cultured porcine islets stained with dithizone. (**E**) Eleven day-cultured porcine islets stained with dithizone. (**F**) Islet organoids on day 11 stained with dithizone (blue arrows).

**Figure 2:**
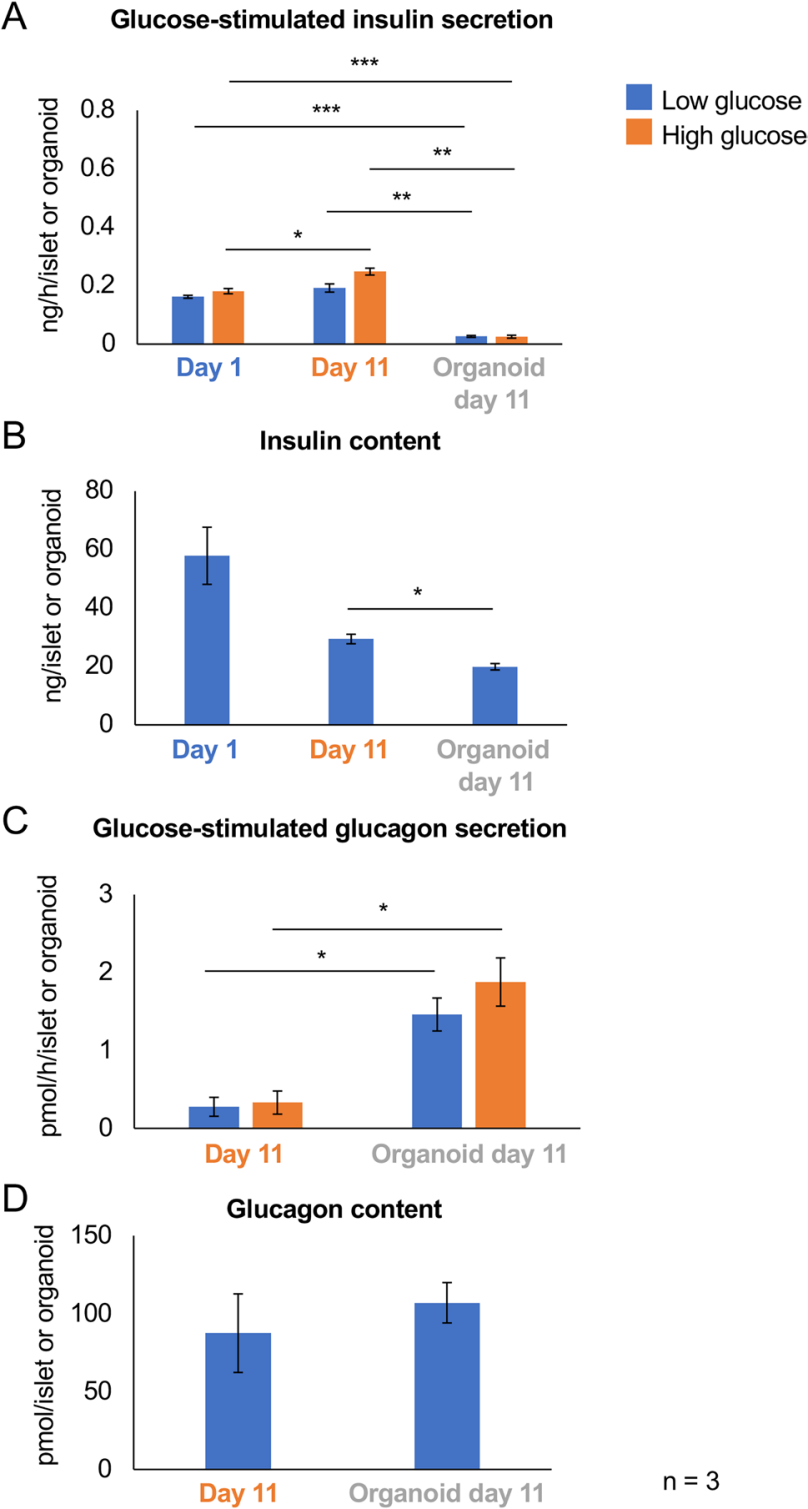
Glucose-stimulated insulin and glucagon secretions of porcine islet organoids on day 11. (**A**) and (**B**) Glucose-stimulated insulin secretion (**A**) and insulin content (**B**) in overnight-cultured islets (Day 1, blue), 11 day-cultured islets (Day 11, orange), and islet organoids (Organoid day 11, gray). (**C**) and (**D**) Glucose-stimulated glucagon secretion (**C**) and glucagon content (**D**) in 11 days cultured islets (Day 11, orange) and islet organoid (Organoid day 11, gray). n = 3. Data are means ± standard error of the median (SEM). *p < 0.05, **p < 0.01, ***p < 0.001.

We elucidated the characteristics of porcine islets in organoid culture. Isolated porcine islets were cultured in advanced DMEM/F12 with Wnt-3A, R-spondin, EGF, Noggin, IGF-1, bFGF, nicotinamide, B27, and some small molecules (organoid culture) or the same medium without these supplements (2D culture) for 11 days (Fig. 1A). Figure 1B shows the shape of islet organoids on day 11. The organoids were obviously larger than overnight- and 11 day-cultured islets (Fig. 1B,D,E). They showed spheroid shapes, including cystic components (Fig. 1B). Organoid viability was high (86.1 ± 4.6%, six islet organoids; Fig. 1C). However, their endocrine function was attenuated compared with overnight- and 11 day-cultured islets. Overnight- and 11 day-cultured islets were stained with dithizone (Fig. 1D and E). However, the dithizone-positive area in islet organoids was limited (Fig. 1F). A significant decrease in glucose-stimulated insulin secretion (GSIS) was observed in islet organoids, but it was preserved in 11 day-cultured islets compared with overnight-cultured islets (0.192 ± 0.014 ng/h/islet vs. 0.026 ± 0.003 ng/h/organoid in low glucose, 0.248 ± 0.012 ng/h/islet vs 0.025 ± 0.005 ng/h/organoid in high glucose, p < 0.01, respectively; Fig. 2A). Insulin content was also decreased in 11 day-cultured islets and islet organoids, particularly islet organoids (29.388 ± 1.624 ng/islet vs. 19.891 ± 1.091 ng/organoid, p < 0.05; Fig. 2B). On the other hand, glucagon secretion of islet organoids was significantly higher comparing with islets in both low and high glucose stimulations (comparison between islets and islet organoids: 0.277 ± 0.122 pmol/h/islet vs. 1.461 ± 0.211 pmol/h/organoid in low glucose, 0.330 ± 0.148 pmol/h/islet vs. 1.878 ± 0.310 pmol/h/organoid in high glucose, p < 0.05 respectively; Fig. 2C). Glucagon contents were 87.436 ± 25.228 pmol/islet in islets vs. 106.940 ± 12.957 pmol/organoid in islet organoids, respectively (Fig. 2D).

**Figure 3:**
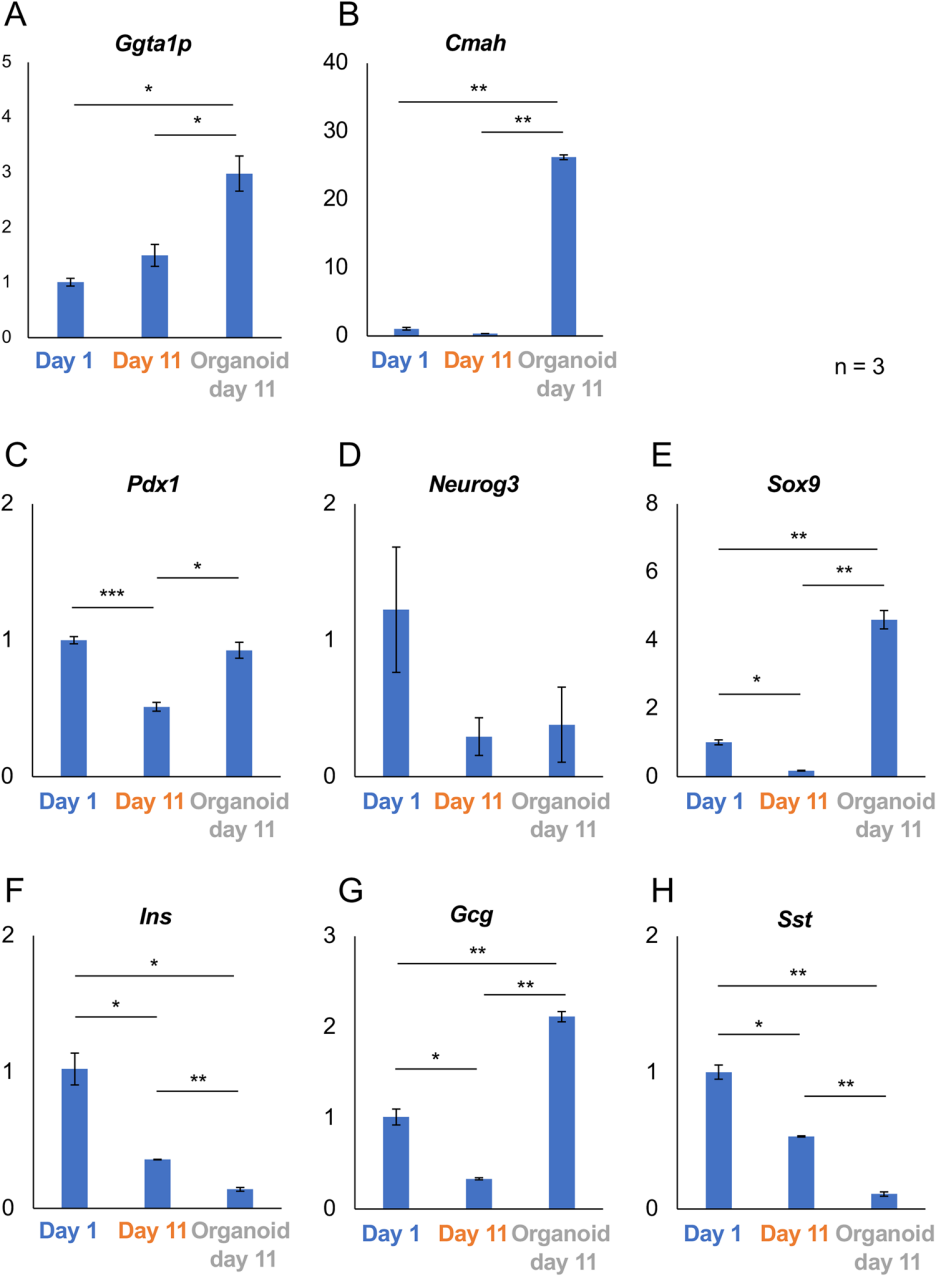
Gene expression in porcine islet organoids on day 11. (**A**–**H**) Expression of genes correlated to carbohydrate antigens (**A**: *Ggta1p*, **B**: *Cmah*), pancreatic differentiation (**C**: *Pdx1*, **D**: *Neurog3*, **E**: *Sox9*), and endocrine hormones (**F**: *Ins*, **G**: *Gcg*, **H**: *Sst*) in overnight-cultured porcine islets (blue), 11 day-cultured islets (orange), and islet organoids on day 11 (gray). The ratios of expression compared with overnight-cultured islets are shown as 2^−ΔΔCt^ values. n = 3. Data are means ± SEM. *p < 0.05, **p < 0.01, ***p < 0.001.

Next, we evaluated gene expression among the three groups. In terms of genes correlated to carbohydrate antigens, expression of *Ggta1p* and *Cmah* in islet organoids was significantly enhanced compared with that in overnight- and 11 day-cultured islets (Fig. 3A and B). In terms of pancreatic differentiation, we assessed the expressions of *Pdx1*, *Ptf1a*, Sox9 and *Neurog3*. *Pdx1* is expressed on multipotent progenitor cells in pancreatic bud. It is required in the earliest step of pancreatic formation. The expression is maintained till the differentiation into β-cells^19^. PTF1 was firstly identified as an acinar enzyme gene activator^20^. As same as *Pdx1*, *Ptf1a* is also expressed in early pancreatic multipotent progenitor cells. After that, *Ptf1a*-expressed multipotent progenitor cells are differentiated into acinar cells, while *Ptf1a* null cells are into bipotent progenitor cells which enable to differentiate into duct/endocrine cells^20^. *Sox9* is also expressed on early pancreatic multipotent progenitor cells^21^. As opposed to *Ptf1a*, the expression of Sox9 is maintained in bipotent progenitor cells and attenuated in acinar cells. Finally, the expression is seen in ductal cells, while disappeared in endocrine progenitors^20^. SOX9 is known as a master regulator of pancreatic differentiation^22^. It acts as a critical maintenance factor of pancreatic progenitors, and contributes to maintenance of pancreatic ducts^23^. Neurogenin 3 work for initiation of the endocrine development. The expression of *Neurog3* is seen on endocrine progenitor^20^. In this study, the expression of *Pdx1* was attenuated in 11 day-cultured islets. However, its expression in islet organoids was recovered to the same level as that in overnight-cultured islets (Fig. 3C). Expression of *Neurog3* tended to be attenuated in 11 day-cultured islets and islet organoids (Fig. 3D). On the other hand, prominent elevation in the expression of *Sox9* was seen in islet organoids (Fig. 3E). Regarding *Ptf1a*, the expression was hardly detected (Ct > 40) in overnight-cultured islets, 11 day-cultured islets and 11 day-cultured islet organoid (data not shown). In terms of islet hormones, attenuation of *Ins* and *Sst* expression and elevation of *Gcg* expression were seen in islet organoids (Fig. 3F–H).

### Long-term cultured islet organoids show the characteristics of pancreatic ducts

**Figure 4:**
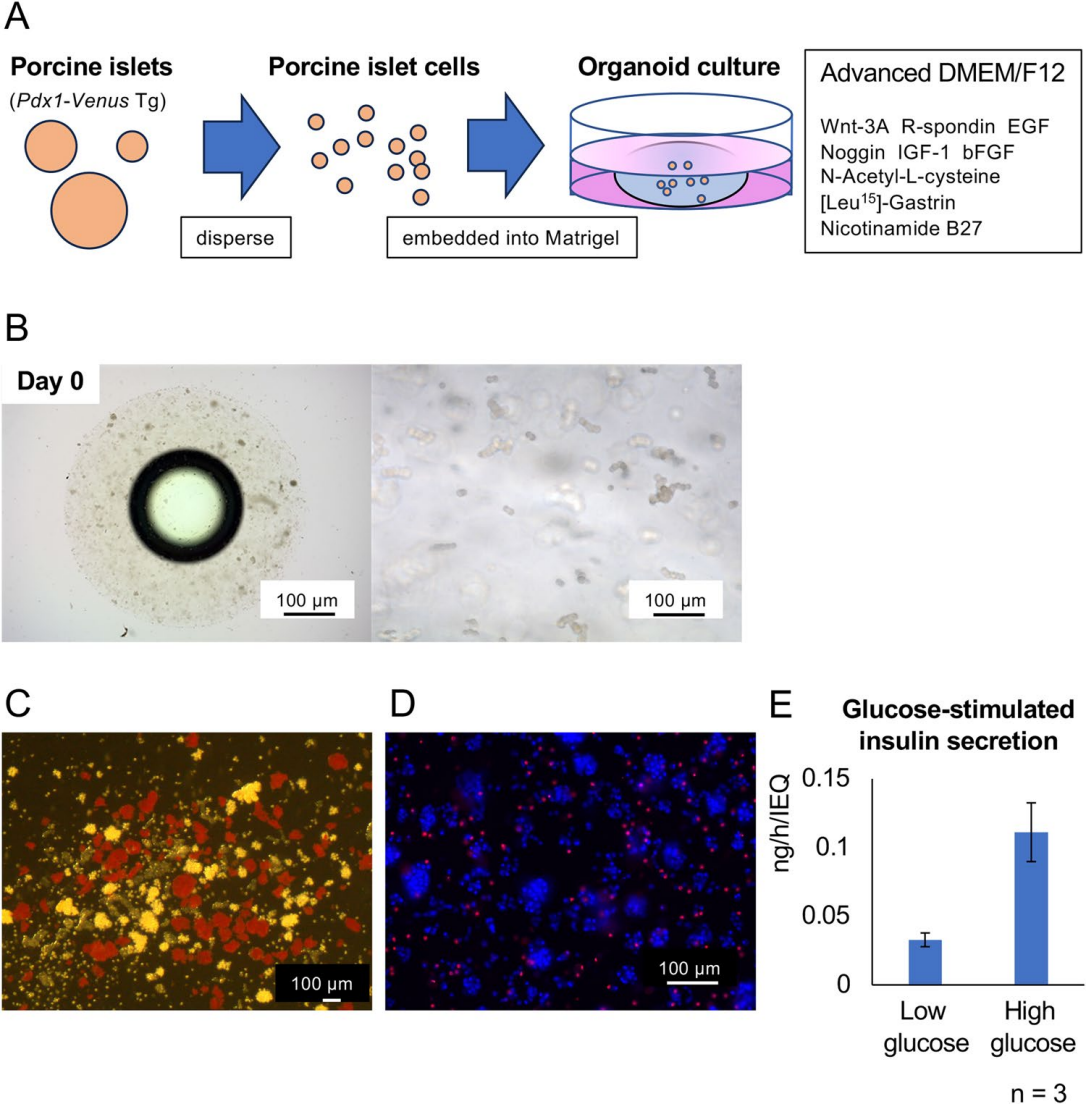
Scheme of porcine islet organoid formation from *Pdx1*-*Venus* Tg pigs. (**A**) Scheme of the formation of islet organoids. Isolated porcine islets from *Pdx1*-*Venus* Tg pigs were dispersed and embedded into growth factor-reduced matrigel. They were cultured in advanced DMEM/F12 with Wnt-3A, R-spondin, EGF, Noggin, IGF-1, bFGF, N-acetyl-L-cysteine, [Leu^15^]-gastrin, and nicotinamide. (**B**) Dispersed islet cells in matrigel [left: low power field (×20, scale bar = 1000 µm), right: high power field (×200, scale bar = 100 µm]. (**C**) Dithizone-stained islets at isolation. Scale bar = 100 µm. (**D**) Overnight-cultured islets stained with Hoechst 33342 (blue) and propidium iodide (red) to assess viability. Scale bar = 100 µm. (**E**) Glucose-stimulated insulin secretion in overnight-cultured islets. n = 3. Data are means ± SEM.

Figure 4A shows the scheme to form islet organoids from dispersed islet cells. Dispersed cells were embedded in growth factor-reduced matrigel and cultured in organoid culture medium (Fig. 4B). This followed an original procedure to form organoids using a biopsy sample from the organ. We assessed the characteristics of islet organoids under long-term culture for > 2 months. Porcine islets were obtained from a transgenic pig whose *Pdx1* gene promotor was conjugated to the *Venus* gene encoding a green fluorescent protein (*Pdx1*-*Venus* Tg pig)^24^. The information of *Pdx1*-*Venus* Tg pig is shown in Table 1. The quality of isolated islets was indicated by 95% cellular purity (Fig. 4C), 93.5 ± 2.0% viability (n = 6 islets, Fig. 4D), and GSIS in accordance with the glucose concentration [low glucose: 0.033 ± 0.005 ng/h/IEQ; high glucose: 0.111 ± 0.021 ng/h/IEQ; stimulation index (ratio of insulin secretion between high and low glucose stimulations): 3.42 ± 0.55; Fig. 4E].

**Table 1:** Characteristics of *Pdx1-Venus* transgenic pigs^24^.

Body weight (kg)	192.5
Trimmed pancreas weight (g)	187
Warm ischemic time (min)	0
Cold ischemic time (min)	471
Islet yields after purification (IEQs)	196,850
Purity after purification on day 1 (%)	95
Viability on day 1 (%)	85

**Figure 5:**
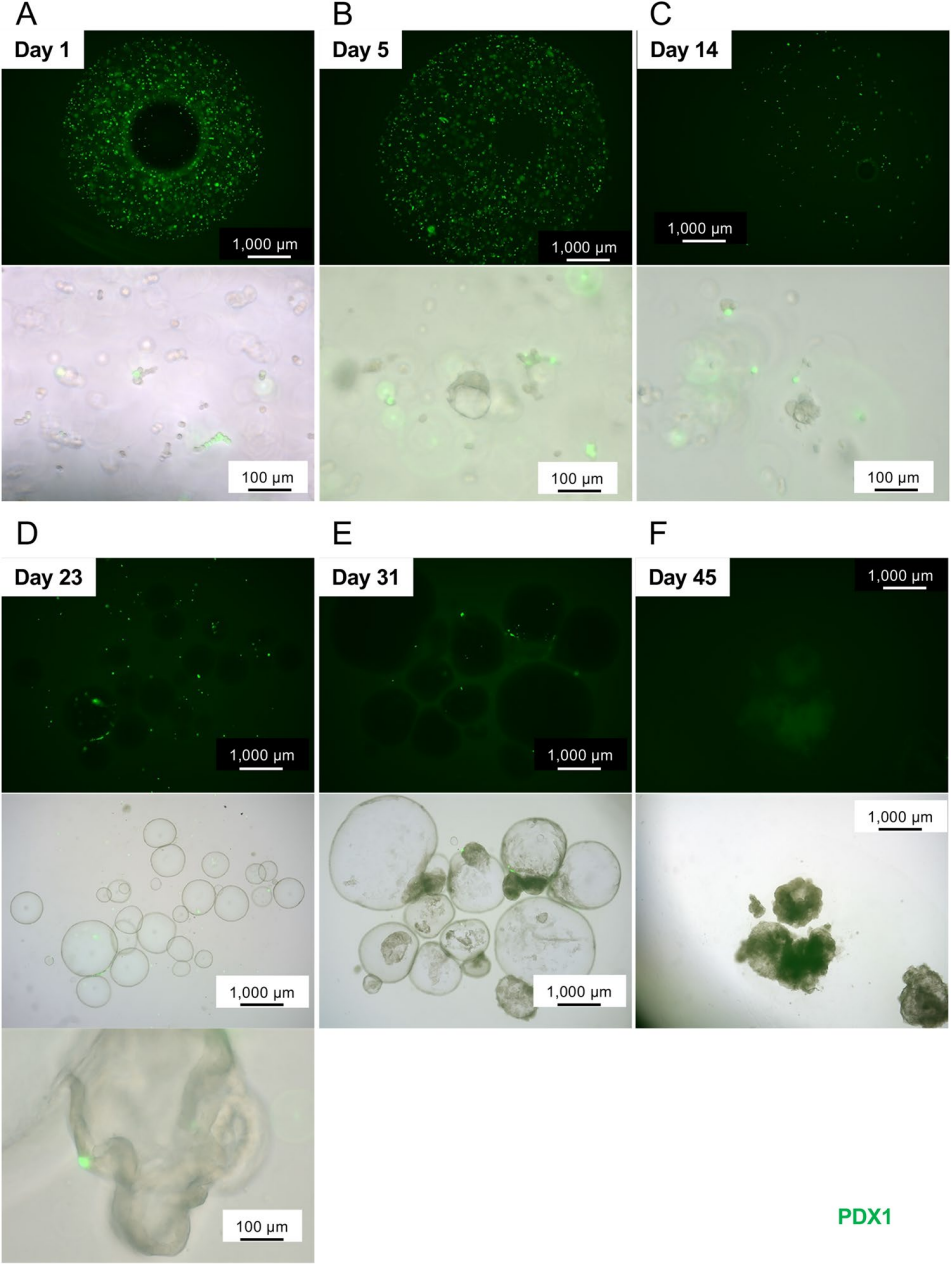
Morphological characteristics of long-term-cultured porcine islet organoids. Dispersed porcine islets isolated from *Pdx1*-*Venus* Tg pigs were embedded in growth factor-reduced matrigel and cultured in organoid medium. (**A**) Day 1, (**B**) day 5, (**C**) day 14, (**D**) day 23, (**E**) day 31, and (**F**) day 45. Upper: fluorescence microscopy images (488 nm excitation), Lower: combination of fluorescence and brightfield microscopy images.

Most dispersed islet cells on day 1 were positive for PDX1 with expression of Venus (Fig. 5A). A few spheroids were seen on day 5. They were positive for PDX1, and some of them included cystic components (Fig. 5B). PDX1-positive spheroids/cells decreased gradually over time, and few positive cells were found on day 14 (Fig. 5C). On day 23, most cells had changed to a cystic construction. The morphological characteristics of islet organoids were similar to those of pancreatic ducts. Some PDX1-positive cells were detected in the cystic construction (Fig. 5D). The cystic components were enlarged and proliferated in the organoid culture (Fig. 5E). On day 45, PDX1-positive cells had completely disappeared (Fig. 5F).

**Figure 6:**
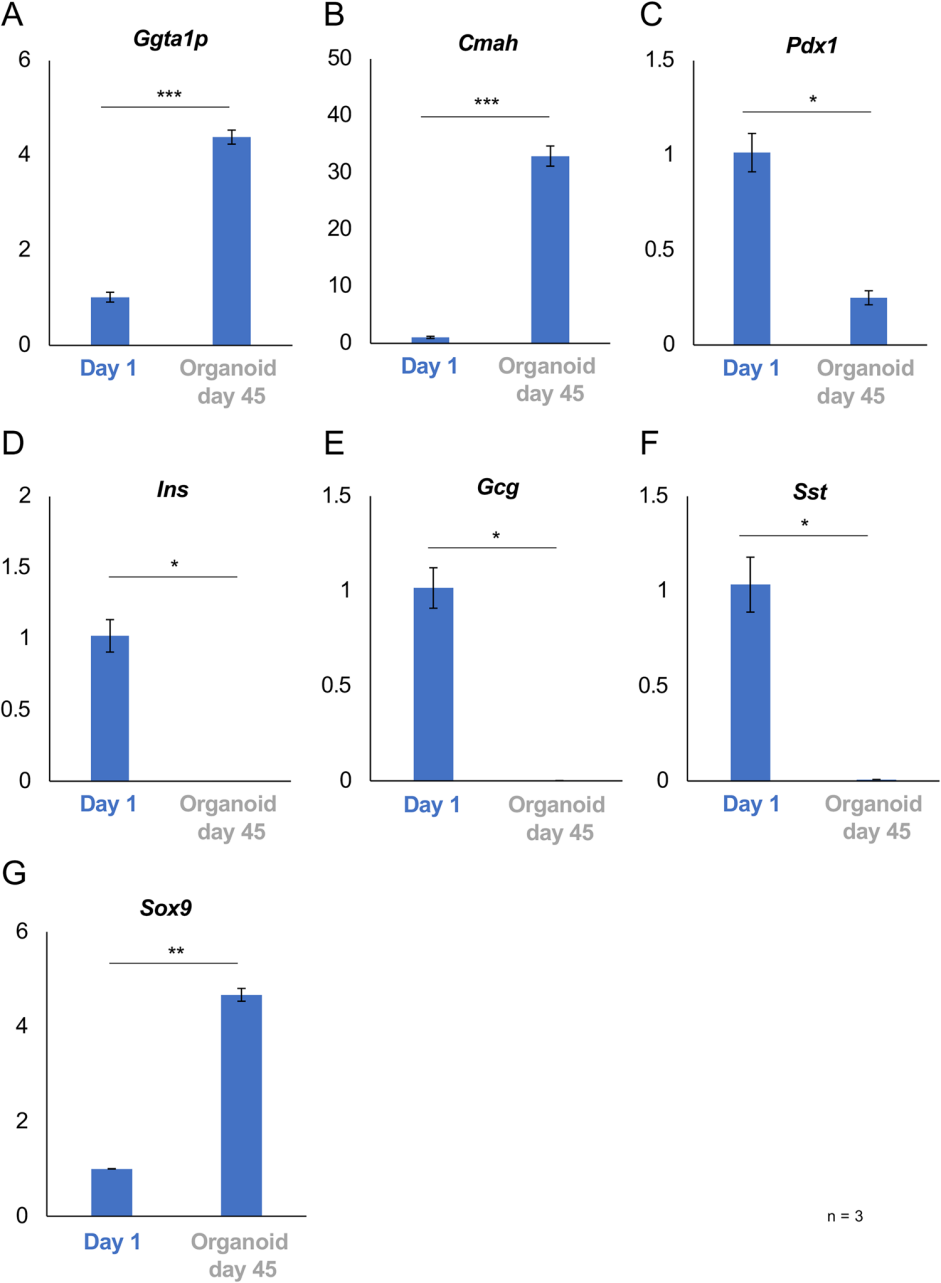
Gene expression in *Pdx1*-*Venus* Tg porcine islet organoids on day 45. (**A**–**G**) Expression of genes correlated to carbohydrate antigens (**A**: *Ggta1p*, **B**: *Cmah*), pancreatic differentiation (**C**: *Pdx1*, **G**: *Sox9*), and endocrine hormones (**D**: *Ins*, **E**: *Gcg*, **F**: *Sst*) in overnight-cultured porcine islets (Day 1, blue) and islet organoids on day 45 (gray). The ratios of the expression compared with overnight-cultured islets are shown as 2^−ΔΔCt^ values. n = 3. Data are means ± SEM. *p < 0.05, **p < 0.01, ***p < 0.001.

Figure 6 shows the gene expression of islet organoids derived from the *Pdx1*-*Venus* Tg pig on day 45 compared with overnight culture. In terms of genes correlated to carbohydrate antigens, significant elevations were seen in *Ggta1p* and *Cmah* expression in islet organoids, as same as islet organoids on day 11 (both p < 0.0001; Fig. 6A and B). In terms of pancreatic differentiation and endocrine hormones, expression of *Pdx1* was significantly decreased and expression of *Ins*, *Gcg*, and *Sst* had completely disappeared on day 45 (all p < 0.05; Fig. 6C–F). Recovery of *Pdx1* expression and elevation of *Gcg* expression on day 11 were cancelled on day 45. However, elevation of *Sox9* expression was also seen on the day (p < 0.01; Fig. 6G).

**Figure 7:**
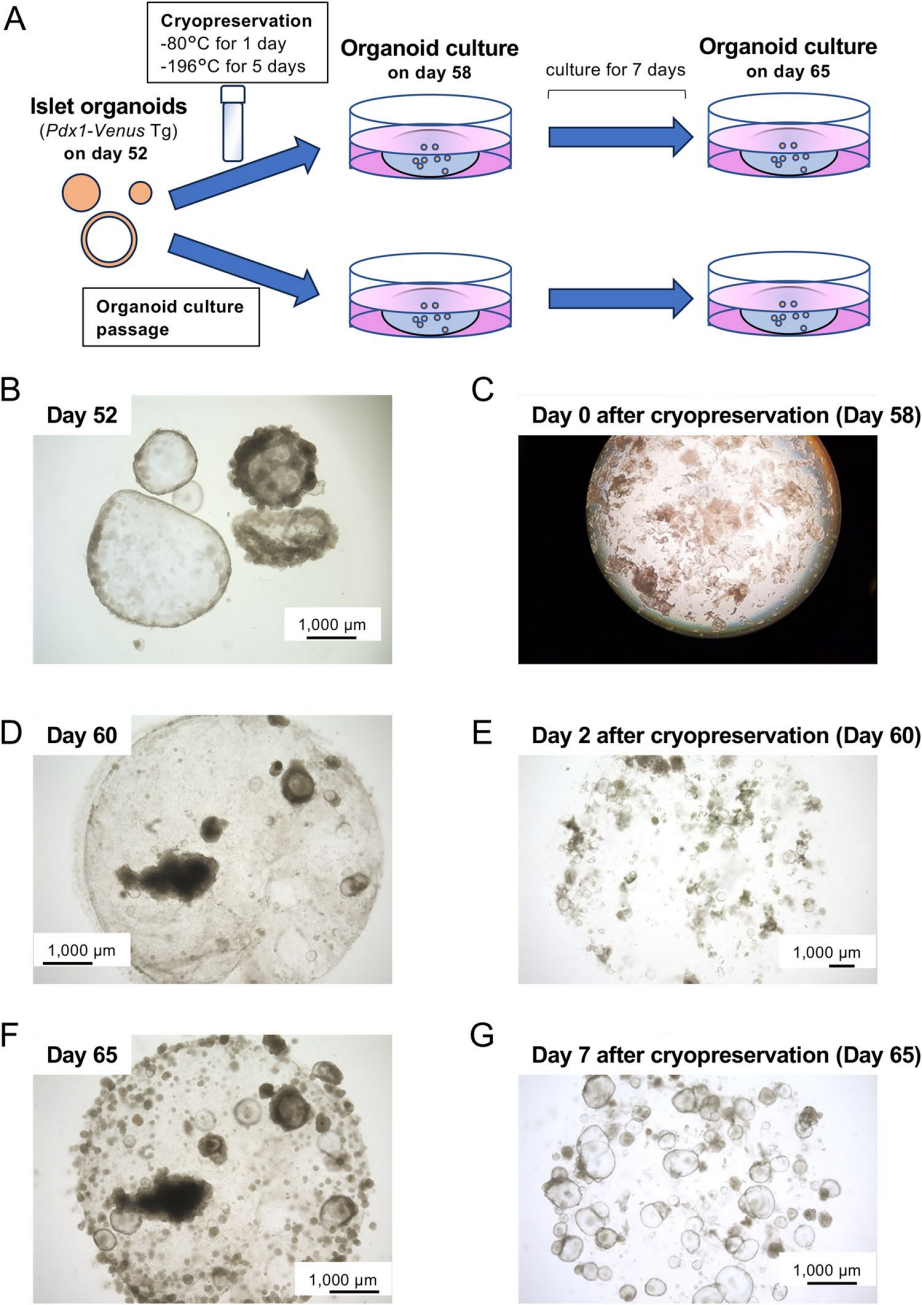
Cryopreservation of porcine islet organoids. (**A**) Scheme of cryopreservation. Porcine islet organoids derived from *Pdx1*-*Venus* Tg pigs were cryopreserved on day 52 using dimethyl sulfoxide (DMSO). They were cryopreserved for 6 days (day 58) and then re-cultured for 7 days (day 65). These organoids were defined as cryopreservation. As a control, residual porcine islet organoids were passaged on day 58 and cultured for 7 days similarly to cryopreservation (defined as organoid culture). (**B**) Islet organoids on day 52. (**C**) Islet organoids after cryopreservation (Day 58). (**D**) Islet organoids on day 60 in organoid culture. (**E**) Islet organoids on day 2 after cryopreservation (Day 60). (**F**) Islet organoids on day 65 in organoid culture. (**G**) Islet organoids on day 7 after cryopreservation (Day 65). Scale bar = 1000 µm.

Next, we performed cryopreservation of some islet organoids on day 52 (Fig. 7B) using Cellbanker® 1 including dimethyl sulfoxide. The islet organoids were cryopreserved for 1 day at − 80 °C and 6 days at − 196 °C. Then, organoid culture was conducted on day 58 (cryopreservation). As a control, organoid culture of residual islet organoids was continued, followed by passaging on day 58 (organoid culture). Organoids were cultured for 7 days (day 65) (Fig. 7A). After cryopreservation, the shape of the cystic component of islet organoids was destroyed (Fig. 7C). After cultivating the organoids, formation of cystic components was seen in both cryopreservation and organoid culture groups on day 2 after cryopreservation/passaging (day 60) (Fig. 7D and E). Then, organoids enlarged and proliferated on day 7 after cryopreservation/passaging (day 65) (Fig. 7F and G).

### Transplanted islet organoids show the characteristics of pancreatic ducts

**Figure 8:**
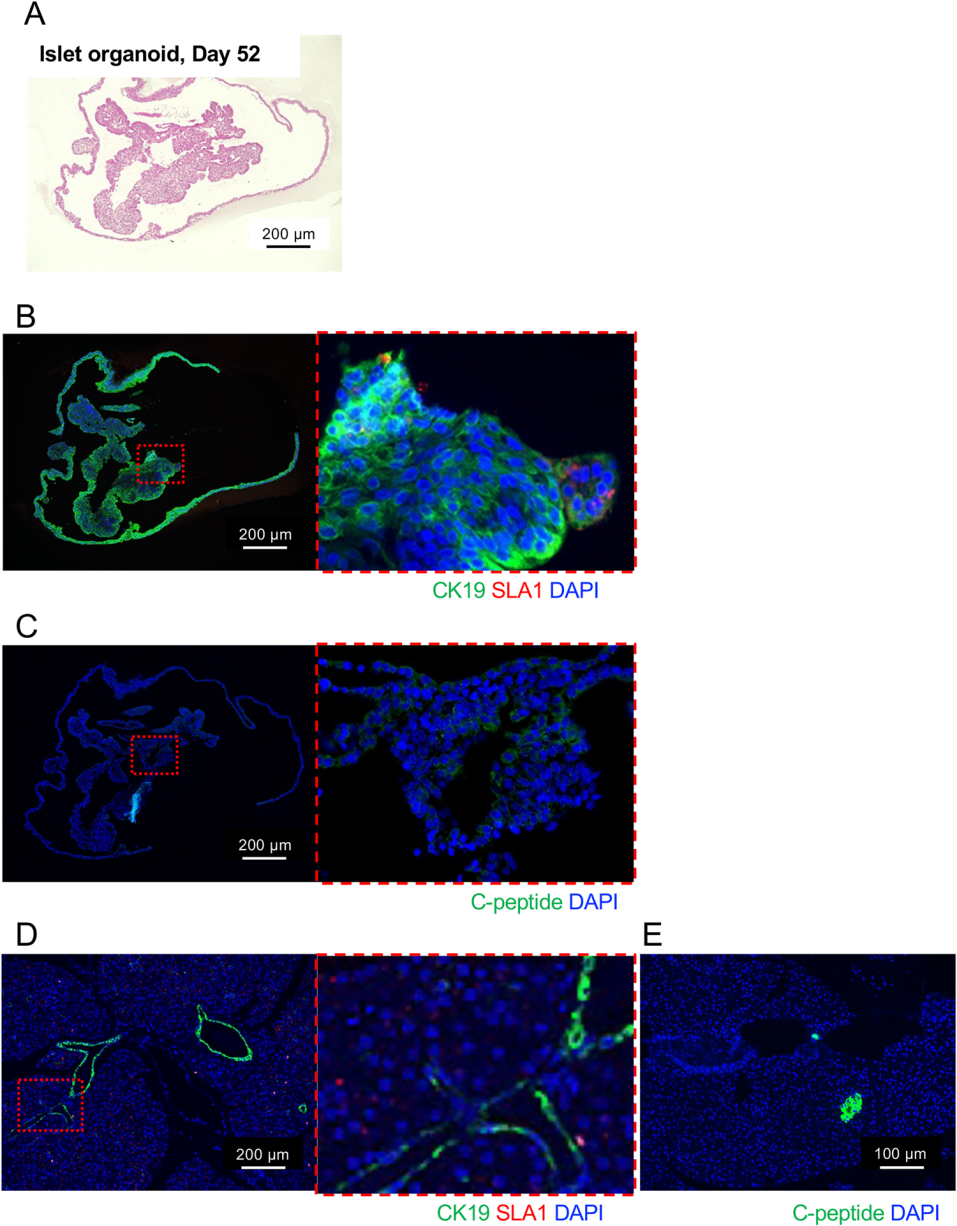
Histological characteristics of islet organoids on day 52. (**A**–**C**) Histological images of islet organoids on day 52 after HE staining (**A**) and immunohistochemical staining of CK19 (green), SLA1 (red) (**B**), and porcine C-peptide (**C**). (**D**) and (**E**) Histological images of porcine pancreas after immunohistochemical staining of CK19 (green), SLA1 (red) (**D**), and porcine C-peptide (**E**). Pancreatic ducts were positive for CK19, and islets were positive for porcine C-peptide. SLA1 was diffusedly stained in the cell membrane of pancreatic cells. Nuclear staining was performed using DAPI (blue). Scale bar = 200 µm.

**Figure 9:**
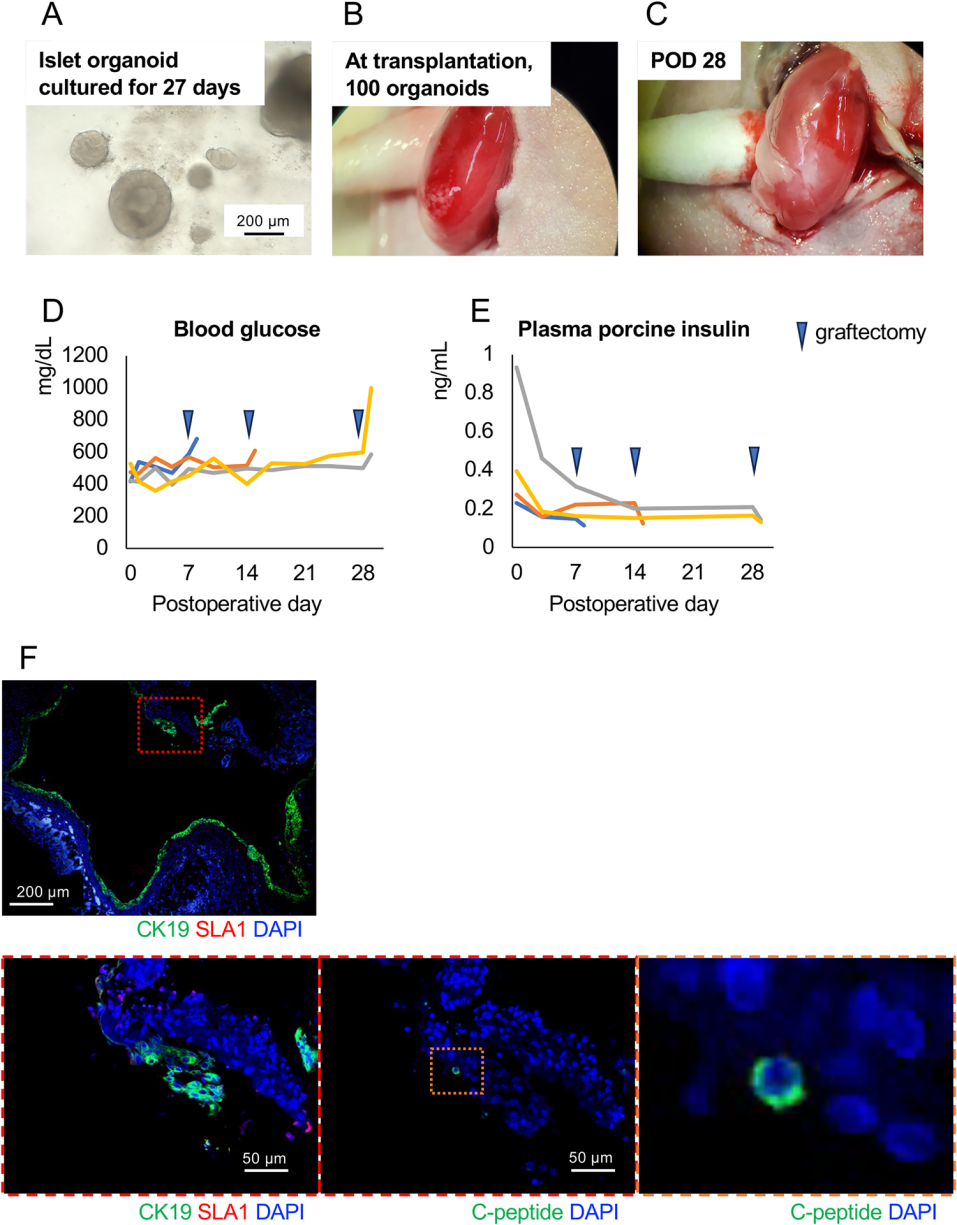
Transplanted islet organoids derived from microminipigs cultured for 27 days. (**A**) Islet organoids derived from microminipigs cultured for 27 days. They showed spheroidal and cystic components. (**B**) Transplanted islet organoids in the subrenal capsular space of diabetic nude mice. (**C**) Engrafted islet organoids on POD 28. (**D**) and (**E**) Blood glucose (**D**) and plasma porcine insulin (**E**) levels after xenotransplantation of 100 islet organoids. Graftectomies were conducted on POD 7 (n = 1), 14 (n = 1), and 28 (n = 2). (**F**) Immunohistochemical staining of engrafted islet organoids on POD 7 for CK19/SLA1 (green and red; upper and lower left, respectively) and porcine C-peptide (green; lower middle and right). Nuclear staining was performed using DAPI (blue). Scale bar = 200 µm (upper) and 50 µm (lower).

We performed xenotransplantation of porcine islet organoids into diabetic nude mice. Prior to transplantation, the characteristics of the islet organoids were assessed histologically. Figure 8A shows a histological image of islet organoids on day 52. They consisted of single and multiple layered columnar cells. Some cells formed rosette shapes. The shapes of nuclei were round and oval. Many of them included nucleoli. All cells comprising islet organoids were positive for CK19. Some of them were positive for SLA1 on the cell membrane (Fig. 8B). However, few cells in the tissues were positive for C-peptide and its expression was mostly weak (Fig. 8C). Figure 9 shows the therapeutic effects of islet organoids derived from microminipigs and cultured for 27 days (Fig. 9A). One hundred islet organoids were xenotransplanted into the renal subcapsular space in diabetic nude mice (Fig. 9B). They were engrafted on POD 28 with feeding blood supplies from recipients (Fig. 9C). No recovery of blood glucose or elevation of plasma porcine insulin was observed after xenotransplantation. A slight increase in blood glucose and decrease in porcine insulin were seen after graftectomy (Fig. 9D and E). Histological assessment revealed that the engrafted islet organoids formed ductal structures. They were positive for CK19 and some were double positive with SLA1. Most of them were negative for porcine C-peptide, and few positive cells were seen (Fig. 9F). These outcomes of islet organoid xenotransplantation coincide the characteristics of these islet organoids, including GSIS, expression of genes correlated with pancreatic differentiation and endocrine hormones, which showed the attenuation of functions as islets.

## Discussion

An organoid is a self-organized 3D tissue derived from pluripotent or tissue stem cells that imitates important functional, structural, and biological characteristics of the original organ^1,25^. Huch and colleagues developed the methodology for pancreatic organoid formation. The organoids were derived from murine pancreatic duct tissue. They found that pancreatic duct tissue cultured in matrigel grew and acquired cystic components with buddings. The pancreatic organoids harbored the characteristics of both pancreatic ducts, including SOX9 expression, and endocrine tissue, including some insulin-positive cells^26^. Subsequently, Azzarelli and colleagues enhanced the β cell characteristics in pancreatic organoids derived from rodent pancreatic ducts by induction of neurogenin 3^27^. Kim et al. developed pluripotent stem cell-derived islet organoids that produced endocrine hormones by induction of differentiation into β cells and the formation of spheroids^28^. Additionally, Wang et al. developed islet organoids that could be passaged and retained the endocrine function long term by co-culturing islet cells with endothelial cells^8^.

In this study, we clarified the characteristics of our porcine islet organoids. Porcine islet-derived organoids that improve the endocrine function of diabetic patients after transplantation might contribute to the promotion of porcine islet xenotransplantation and curing severe diabetes. We assessed the characteristics of relatively short-term-cultured porcine islet organoids (11 days). The organoid culture medium contained Wnt, R-spondin, and Noggin. R-spondin is an enhancer of Wnt/β-catenin that promotes cellular proliferation and maintains the differentiation behavior of stem cells^29^. Noggin is an extracellular antagonist of bone morphogenetic protein and plays an important role in the formation of neural, intestinal, hepatic, and pulmonary organoids^30^. These factors are required to maintain the condition of stem cells in porcine islets. On day 11, the porcine islets had enlarged compared with the original porcine islets, indicating that cell proliferation was promoted by the organoid culture, particularly the supplements. Islets are a well-differentiated tissue with poor cellular proliferation. Enhanced cell proliferation is considered to be a preferable characteristic for stocking because cell proliferation after cryopreservation recovers the condition of organoids injured by cryopreservation. However, islet organoids, including cystic/ductal structures, had an attenuated endocrine function. Our islet organoids secreted insulin under glucose stimulation. However, this function was significantly inferior to that of islets on day 11. Conversely, glucagon secretion of islet organoids was significantly higher than islets on the day. Recovery of *Pdx1*, elevation of *Sox9* and *Gcg* expression, and attenuation of *Ins* and *Sst* expression were seen in the islet organoids. These results indicated that relatively short-term-cultured islet organoids showed the characteristics of pancreatic ducts, but had a partially preserved endocrine function (insulin and glucagon secretions, recovery of *Pdx1* expression, and elevation of *Gcg* expression).

Next, we implemented long-term (45 days) organoid culture of *Pdx1*-*Venus* Tg porcine islets. This model was clearly suitable to follow PDX1-positive tissue considered to be islets. Most islet cells were positive at 1 week, and cystic and ductal structures were seen during this time. On day 14, PDX1-positive tissue was significantly decreased. On days 23 and 31, islet organoids with a cystic structure were enlarged and proliferated with attenuation of PDX1 expression. On day 45, PDX1-positive tissue had completely disappeared. The tendency of gene expression in islet organoids was similar to that on day 11. However, both recovery of *Pdx1* expression and elevation of *Gcg* expression seen on day 11 were completely cancelled on day 45. *Sox9* expression was also seen in the islet organoids. Furthermore, expression of carbohydrate antigen-correlated genes (*Ggta1p* and *Cmah*) was strongly elevated in islet organoids. Thus, long-term-cultured islet organoids harbored similar characteristics to pancreatic ducts and the characteristics of endocrine tissue had completely disappeared. The organoid culture medium contained B27 and nicotinamide, which contribute to β cell proliferation^31,32^, but attenuation could not be prevented in this study. It was also revealed that the islet organoids could be cryopreserved in this study, but the characteristics were likely those of pancreatic ducts.

**Figure 10:**
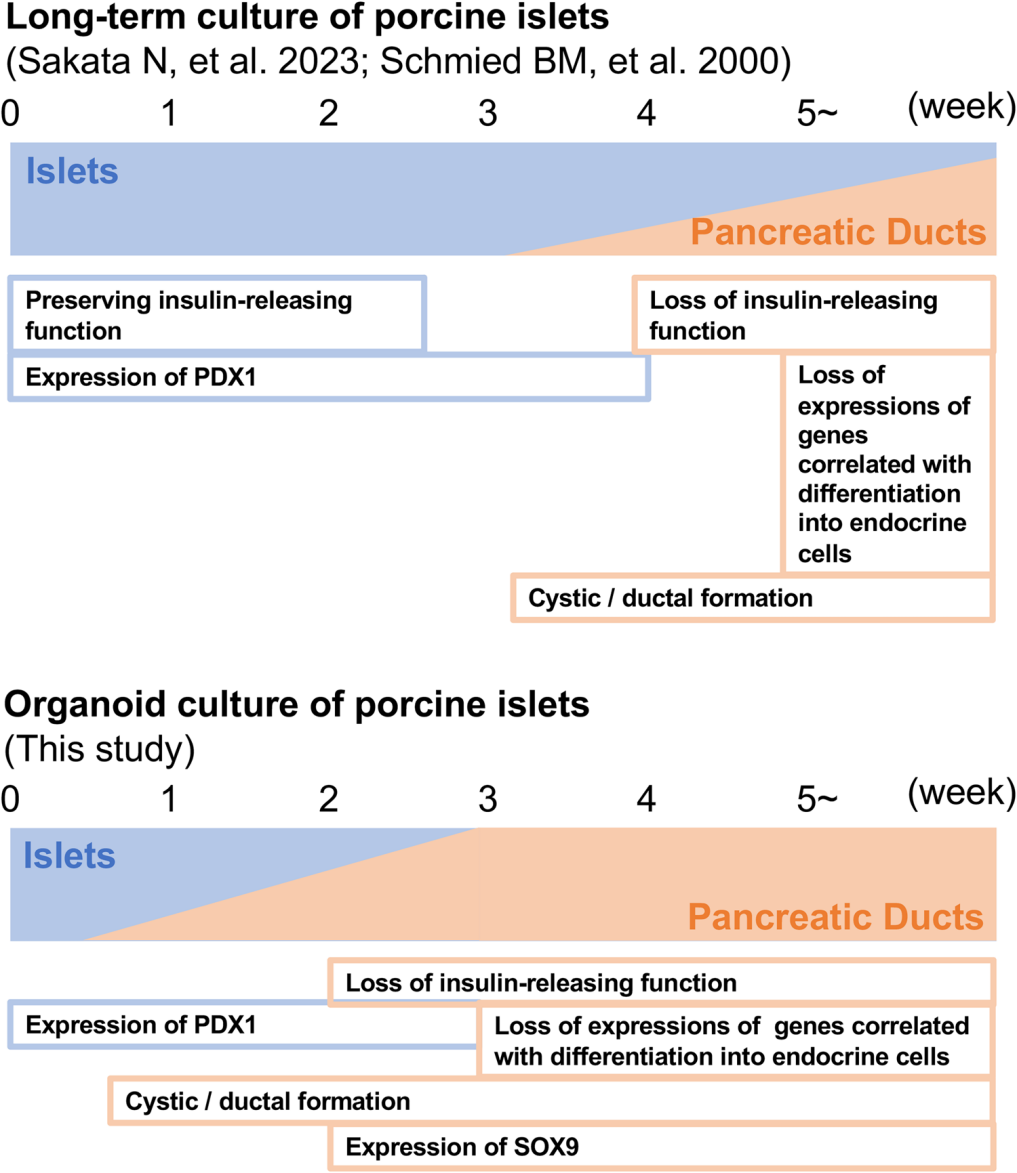
Events in porcine islets correlated to preservation of islets and differentiation into pancreatic ducts seen in long-term culture (upper) and organoid (lower) culture.

We considered that these consequential events of porcine islets seen under organoid culture resembled those of long-term-cultured porcine islets (Fig. 10). Our previous study revealed that porcine islets cultured for 28 days acquire cellular proliferation and partial recovery of the endocrine function at 37 °C. The long-term culture stressed porcine islets, but they resisted the stress by incubation at 37 °C. We considered that islet stem cells, including pancreatic stellate cells, might contribute to islet regeneration under our culture conditions^15^. However, 28 days might be the time limit for islet regeneration. Schmied and colleagues showed that ductal formation begins in cultured hamster islets on day 7, which proliferate gradually. On day 35, undifferentiated ductal cells expressing cytokeratin and antitrypsin, and not expressing insulin-promoting factor 1, NKx6.1, Pax6, or NeuroD correlated islet development^33^. Furthermore, they found that 60 day-cultured human islets changed into undifferentiated ductal cells expressing cytokeratin 7 and 19^34^. These data showed that long-term culture for > 1 month might convert islets into ductal cells. Moreover, organoid culture might promote such conversion.

Our study revealed that used supplements for promoting organoid formation did not contribute to maintain the characteristics as islet. Among the supplements, we used EGF, nicotinamide and B27, which played a role in inducting differentiation into insulin-producing cells^35^. It might be necessary to use further supplements, such as activin A, retinoic acid, for mainraining characteristics as islet^36^.

In summary and conclusion of this study, we elucidated the characteristics of porcine islet organoids derived from porcine islets. Porcine islet organoids proliferate and can be cultured and passaged for a long time and cryopreserved. However, they show the characteristics of pancreatic ductal cells. These changes were seen in long-term-cultured porcine islets. Therefore, the formation of porcine islet organoids imitates the changes in porcine islets under long-term culture. Futher study is necessary for producing porcine islet-derived organoids having characteristics as islets.

## Materials and methods

### Animals

As donor animals, microminipigs (Fuji Micra Inc., Fujinomiya, Japan) and *Pdx1*-*Venus* Tg pigs (kindly provided by Prof. Hiroshi Nagashima, Meiji University) were used. Eight to 12-week-old BALB/cAJcl-*nu*/*nu* male mice (CLEA Japan Inc., Tokyo, Japan) were also used as recipients for organoid transplantation. Animals were housed under specific pathogen-free conditions with free access to food and water. The care of mice and experimental procedures complied with the ‘‘Principles of Laboratory Animal Care’’ [Guide for the Care and Use of Laboratory Animals, 8th edition (National Research Council, 2011)]. The experimental protocol was approved by the Animal Care and Use Committee of Fukuoka University (Approval number: 2112093).

### Pancreas collection

The porcine pancreas was harvested as described in our previous studies^15,37^. In brief, a total pancreatectomy was performed under general anesthesia using isoflurane. After heparinization, the pigs were exsanguinated by incising the vena cava in the thoracic cavity, and Belzer UW^®^ Cold Storage Solution (https://amn.astellas.jp/content/dam/jp/amn/jp/ja/di/pdf/blz/Belzer_UW_Cold_Storage_Solution.pdf; Preservation Solutions, Inc. Elkhorn, WI) was infused via the abdominal aorta, while abdominal organs were cooled using crushed ice. After flushing out blood, the total pancreatomy was performed. An 18–24 G intravenous catheter was inserted into the pancreatic duct, and cold preservation solutions (ET-Kyoto solution; Cat# 035-13121-2; Otsuka Pharmaceutical Factory, Inc., Naruto, Japan, and ulinastatin; Cat #3999405A2077; Mochida Pharmaceutical Co., Tokyo, Japan) were infused at 1 mL/g pancreas mass.

### Porcine islet isolation

Porcine islet isolation was also conducted as described in our previous studies^15,37^. A collagenase solution containing liberase MTF (0.5 g per 1 vial) and thermolysin (15 mg per 1 vial) (Cat# 05339880001; Roche CustomBiotech, Penzberg, Germany) was instilled into the disinfected pancreas via the catheter placed in the pancreatic duct. The distended pancreas was placed in a Ricordi chamber. The digestion process was initiated by shaking the chamber to circulate the warmed collagenase solution. After stopping digestion and washing the digested pancreatic tissue with RPMI 1640 medium (Cat# 11875085; Thermo Fisher Scientific, Gibco, Waltham, MA) containing 10% inactivated plasma (fetal bovine serum, qualified, United States, Cat #26140079; Thermo Fisher Scientific, Gibco), the tissues were collected in Belzer UW^®^ Cold Storage Solution. The purification process was performed using IBM 2991 (COBE 2991; Terumo BCT, Tokyo, Japan) by centrifugation with a continuous density gradient between 1.077 and 1.100 g/cm^3^ created using Optiprep (Cat# ST-07820; Veritas Co., Tokyo, Japan). After centrifugation, gradient density solutions containing highly purified islets (≥ 70%) were collected.

### Dispersion of islets into single cells

Isolated islets were dispersed into single islet cells using Accutase (Cat# 12679-54; Nacalai Tesque, Kyoto Japan) by warming at 37 °C for 25 min. Then, the dispersed cells were washed three times with Hank’s balanced salt solution (Cat# 14025092; Thermo Fisher Scientific) containing 0.2% bovine serum albumin (Cat #A9418; Merck, Sigma-Aldrich, St. Louis, MO).

### Formation of islet organoids

Islets or dispersed islet cells were suspended on ice at approximately 5 × 10^5^ cells/50 µL in liquidized growth factor-reduced matrigel (Cat# 356231; Corning, Corning, NY). The suspended cells were seeded on a well in a 24-well plate. After gelation by warming at 37 °C, 500 µL culture medium was added to the well. The culture medium was 5 mL advanced DMEM/F12 (Cat# 12634010; Thermo Fisher Scientific) with 1% GlutaMax Supplement (Cat# 35050061; Thermo Fisher Scientific), 5 mL Wnt-3A conditioned medium (Cat# J-ORMW301R; MBL Life Science, Tokyo, Japan), 1 µg/mL recombinant mouse R-spondin (Cat#3 474-RS; R&D Systems, Minneapolis, MN), 500 ng recombinant mouse epidermal growth factor (EGF) (Cat# PMG8041; Thermo Fisher Scientific), 1 µg recombinant mouse Noggin (Cat#250-38; Thermo Fisher Scientific, PeproTech), 1 µg recombinant insulin-like growth factor 1 human (IGF-1) (Cat# 590906; BioLegend, San Diego, CA), 500 ng recombinant human basic fibroblast growth factor (bFGF) (Cat# 100-18B; Thermo Fisher Scientific, PeproTech), 500 nM A83-01 (Cat# 039-24111; FUJIFILM Wako Pure Chemical, Osaka, Japan), 10 µM Y-27632 (Cat# 030-24021; FUJIFILM Wako Pure Chemical), 200 µL B27 supplement (Cat# 17504044, Thermo Fisher Scientific), 1 mM N-acetyl-l-cysteine (Cat# A9165-5G, Merck), 10 nM [Leu^15^]-gastrin I human (Cat# G9145-.1MG, Merck), and 10 mM nicotinamide (Cat# N0636-100G, Merck). The cells were cultured at 37 °C with 5% CO_2_. The medium was changed every 3 or 4 days. Morphological changes of the organoids were recorded under a BZ-X700 microscope (Keyence, Itasca, IL). Organoid passaging was conducted every 7–10 days when the proliferating organoids reached confluency in the matrigel.

### Cryopreservation of islet organoids

We attempted to cryopreserve the organoids because islets, especially porcine islets, are difficult to cryopreserve because of their vulnerability and maturity. Organoids were suspended in 1 mL Cellbanker 1 (Zenogen Pharma, Koriyama, Japan) and gradually cooled to − 80 °C using a BICELL (Nihon Freezer Co., Tokyo, Japan) to prevent cellular damage. After overnight cooling, the organoids were cryopreserved in liquid nitrogen. Frozen organoids were thawed by warming at 37 °C.

### Glucose-stimulated insulin and glucagon secretions

Glucose-stimulated insulin secretion (GSIS) and glucagon secretion (GSGS) of porcine islets and islet organoids and islets was assessed by treatment with various concentrations of glucose. In brief, 300 islet equivalents (IEQs) and organoids were preincubated with 3.3 mM glucose for 60 min. After preincubation, the islets were stimulated with glucose at 3.3 mM (low glucose) or 16.5 mM (high glucose) for 60 min. Insulin and glucagon in culture supernatants were measured using an LBIS Porcine Insulin enzyme-linked immunosorbent assay (ELISA) Kit (Fujifilm Wako Shibayagi Co., Shibukawa, Japan) and a Glucagon ELISA Kit (Wako), respectively.

### Measurement of insulin and glucagon contents

Internal insulin and glucagon were extracted from 300 IEQs and organoids using 1 mL RIPA buffer (Cat#16488-34; Nacalai Tesque, Kyoto, Japan) containing × 100 protease and phosphatase inhibitor cocktails (Cat#07575-51 and Cat#07574-61; Nacalai Tesque). The insulin and glucagon contents were measured using an LBIS Porcine Insulin ELISA Kit and a Glucagon ELISA Kit (Wako), respectively.

### Cell viability of islet organoids

Islet organoids were stained with Hoechst^®^ 33342 (viable) and propidium iodide (PI; dead) (Thermo Fisher Scientific K.K., Tokyo, Japan). Cell viability of islets was defined as the percentage of Hoechst^®^ 33342-positive cells per total endocrine cells in an islet ([Hoechst^®^ 33342-positive cells]/([Hoechst^®^ 33342-positive cells] + [PI-positive cells]) × 100).

### Real-time reverse transcription-polymerase chain reaction analysis

RNA was extracted from porcine islet samples using TRIzol (Cat# 15596026; Thermo Fisher Scientific, Invitrogen) and purified using a PureLink^®^ RNA Mini Kit (Cat# 12183018A; Thermo Fisher Scientific) in accordance with the manufacturers’ instructions. RNA concentrations were equalized using a NanoDrop 2000 spectrophotometer (Thermo Fisher Scientific). Reverse transcription was performed using a QuantiTect Reverse Transcription Kit (Cat# 205311; Qiagen K.K., Tokyo, Japan). qRT-PCR analysis was performed using a CFX Connect Real-Time PCR Detection System (Bio-Rad Laboratories, Inc., Hercules, CA) and Thunderbird SYBR qPCR Mix (Cat# QPS-101: Toyobo Co., Ltd., Osaka, Japan). The primers used for real-time RT-PCR are shown in Table 2. Primers were designed by Fasmac Co., Ltd. (Atsugi, Japan). Relative quantitation was performed using LightCycler Software Version 4.1. The results were normalized to expression of a reference gene (*Actb*). Data are presented as the fold difference calculated using the 2^−ΔΔCt^ method.

**Table 2:** Porcine primers for real-time reverse transcription-polymerase chain reaction analysis.

Primer name	Sequence (5′-3′)	Tm (°C)
*Actb*_F	CTCCAGAGCGCAAGTACTCC	60.18
*Actb*_R	TGCAGGTCCCGAGAGAATGA	60.61
*Ggta1*_F	GAAACCCAGAAGTTGGCAGC	59.68
*Ggta1*_R	CAGTCCACTAGCGGAAGCTC	60.18
*Cmah*_F	TCACATGCACTCAGACCACC	59.96
*Cmah*_R	CAACTGGACGCCACTCTGAT	60.04
*Ins*_F	GGCTTCTTCTACACGCCCAA	60.32
*Ins*_R	GCGGCCTAGTTGCAGTAGTT	60.39
*Gcg*_F	GATCATTCCCAGCTCCCCAG	59.89
*Gcg*_R	GTGTTCATCAGCCACTGCAC	59.76
*Sst*_F	CCCGACTCCGTCAGTTTCTG	60.39
*Sst*_R	GGCATCGTTCTCTGTCTGGT	59.75
*Pdx1*_F	AAGTCTACCAAGGCTCACGC	60.04
*Pdx1*_R	GCGCGGCCTAGAGATGTATT	60.04
*Neurog3*_F	CTCTATCCCTCAGCGCCCTA	60.25
*Neurog3*_R	CGACGCAGGTCACTTTGTCT	60.60
*Ptf1a*_F	GGCCATCGGCTACATCAACTT	60.75
*Ptf1a*_R	ATAATCCGGGTCGCTGGGA	60.46

### Induction of diabetes in recipient mice

Diabetes was induced in recipient mice by intravenous injection of streptozotocin (220 mg/kg body weight; Sigma-Aldrich). Mice with blood glucose levels exceeding 400 mg/dL were used as diabetic recipients.

### Organoid transplantation

Recipient mice were anesthetized using isoflurane. A dorsal incision was made through the muscle and peritoneum, and the left kidney was mobilized outside the abdomen. The renal capsule was peeled off from the parenchyma to prepare the renal subcapsular space for transplantation of islet organoids. Porcine islet organoids were placed in the space. After transplantation, the kidney was placed back in the abdomen, and the incision was sutured.

The function of transplanted islets was assessed by monitoring blood glucose and plasma insulin concentrations. Plasma concentrations of porcine insulin were measured using an LBIS Porcine Insulin ELISA Kit (Fujifilm Wako Shibayagi Co.).

### Histological assessment

Left kidneys of the recipient mice were dissected out under general anesthesia and fixed using 10% formalin. Then, the kidney was embedded in paraffin. Three-micrometer-thick sections were either stained with hematoxylin and eosin (HE) or subjected to immunohistochemistry of porcine C-peptide to identify porcine islets, CK-19 to detect pancreatic ductal components, and swine leukocyte antigen class I (SLA I) to detect porcine-derived tissue. The primary antibodies were mouse anti-pig C-peptide (1:200; Cloud-Clone Corp. MAA447Po21, Katy, TX), rabbit anti-KRT19/CK19/cytokeratin 19 polyclonal (1:100; LSBio. LS-B13606-50, Shirley, MA), and mouse anti-pig SLA I (1:100; Bio-Rad Laboratories Inc., MCA2261PE, Hercules, CA) antibodies. After incubation with a primary antibody, donkey anti-mouse IgG (H + L) Alexa 488 (1:100; Jackson ImmunoResearch Laboratories, Inc., West Grove, PA,) or Cy3-conjugated goat anti-rabbit (1:100; Jackson ImmunoResearch Laboratories, Inc.) was used as a secondary antibody. Nuclear staining was performed using 4′,6-diamidino-2-phenylindole (DAPI). Histological images were obtained under the BZ-X700 microscope.

### Statistical analysis

The unpaired t-test or Dunnett’s test was used for paired and multiple comparisons, respectively. Data are presented as the mean ± standard error of the mean. p < 0.05 was used to define statistical significance. All tests were two-sided. Statistical analyses were conducted using JMP^®^12.0.0 (SAS Institute Inc., Cary, NC).

### Statement on ARRIVE guidelines

This study was reported in accordance with the ARRIVE guidelines. All experiments were performed in accordance with relevant guidelines and regulations.

## Data Availability

The datasets generated during the current study are available from the corresponding author on reasonable request.
